# Transcriptional expressions of hsa-mir-183 predicted target genes as independent indicators for prognosis in bladder urothelial carcinoma

**DOI:** 10.18632/aging.204040

**Published:** 2022-05-03

**Authors:** Ming Li, Da-Ming Xu, Shu-Bin Lin, Zheng-Liang Yang, Teng-Yu Xu, Jin-Huan Yang, Ze-Xin Lin, Ze-Kai Huang, Jun Yin

**Affiliations:** 1 Division of Urological Surgery, Second Affiliated Hospital of Shantou University Medical College, Shantou, Guangdong, China; 2 Division of Hematology, Second Affiliated Hospital of Shantou University Medical College, Shantou, Guangdong, China; 3 Department of Clinical Laboratory Medicine, Second Affiliated Hospital of Shantou University Medical College, Shantou, Guangdong, China

**Keywords:** hsa-mir-183, predicted target gene, TP53, prognosis, bladder urothelial carcinoma

## Abstract

Objective: To uncover novel prognostic and therapeutic targets for BLCA, our study is the first to investigate the role of hsa-mir-183 and its up-regulated predicted target genes in bladder urothelial carcinoma.

Methods: To address this issue, our study explored the roles of hsa-mir-183 predicted target genes in the prognosis of BLCA via UALCAN, Metascape, Kaplan-Meier plotter, Human Protein Atlas, TIMER2.0, cBioPortal and Genomics of Drug Sensitivity in Cancer databases.

Results: High transcriptional expressions of PDCD6, GNG5, PHF6 and MAL2 were markedly relevant to favorable OS in BLCA patients, whereas SLC25A15 and PTDSS1 had opposite expression significance. Additionally, high transcriptional expression of PDCD6, GNG5, PHF6, MAL2, SLC25A15 and PTDSS1 were significantly correlated with BLCA individual cancer stages and molecular subtypes. Furthermore, high mutation rate of PDCD6, MAL2, SLC25A15 and PTDSS1 were observed. Finally, TP53 mutation of PDCD6, GNG5, PHF6, MAL2, SLC25A15 and PTDSS1 has guiding significance for drug selection in BLCA.

Conclusions: PDCD6, GNG5, PHF6, MAL2, SLC25A15 and PTDSS1 could be the advanced independent indicators for prognosis of BLCA patients, and TP53-mutation might be a biomarker for drug option in BLCA patients.

## INTRODUCTION

Bladder Urothelial Carcinoma (BLCA) is one of the most common urinary system tumors with the unfavorable mortality and increased incidence in the world [1, 2]. The histological features of BLCA are the transformation between urothelium and malignant cells, Involving a series process of polymorphic nuclei, hyperchromatic nuclei, nucleolar herniation, and an increased number of uncontrolled mitoses [3]. Recently, the detailed molecular subtypes of BLCA have been widely explored, namely neuronal, basal squamous, luminal, luminal infiltrated and luminal papillary, which has important prognostic and therapeutic implications. Studies on BLCA have suggested that BLCA *in situ* without treatment may lead to progression to muscular invasive disease (occur in 50%) and disease relapse (occur in 90%) [4, 5]. New insights on the pathogenesis, invasion and metastasis of BLCA have been ongoing in recent studies, however, the research on the gene molecular basis of bladder cancer has been limited. Providing crucial insight into the gene level characteristics of BLCA will help to better reveal the mechanism of pathogenesis and discover more significant diagnostic or prognostic biomarkers and therapeutic options.

MicroRNA (miRNA), a small noncoding RNA with the function of negative regulating gene expression, plays an important role in the occurrence and development of various tumors [6–8]. The functions of miRNA are not to directly block the expression of genes, but to make a slightly and specifically adjustment to the expression of target genes in post-transcriptional control [9, 10]. MiRNA plays an important regulatory role in the biological processes of normal human cells, including cell division cycle, cell growth, cell apoptosis, cell differentiation and cell response. Almost all tumors have different types of miRNA expression, which are either up-regulated or down-regulated, thus providing new opportunities for miRNA and their predicted target genes to be used as the potential diagnostic or prognostic biomarkers. Hsa-mir-183 has been shown to frequently involve methylation events in human hepatocellular carcinoma and was an unfavorable factor for survival [11]. Huang et al., found that hsa-mir-183 was an overexpressed miRNA in non-small-cell lung cancer and was positively correlated with tumor invasiveness [12]. Additionally, hsa-mir-183 has been reported to be markedly correlated with overall survival for colon adenocarcinoma patients [13]. However, the role of hsa-mir-183 and its predicted target genes in BLCA remains unclear.

To uncover novel prognostic and therapeutic targets for BLCA, our study is the first to investigate the role of hsa-mir-183 and its up-regulated predicted target genes and evaluate the significance of TP53-mutation related drug option in BLCA patients.

## MATERIALS AND METHODS

To estimate the prognostic and therapeutic significance of hsa-mir-183 and its up-regulated predicted target genes in BLCA patients, the clinical data, transcriptional expression data of miRNA and predicted target genes were collected from UALCAN (http://ualcan.path.uab.edu). The Metascape was applied for GO and KEGG analysis of predicted target genes (https://metascape.org). The Overall Survival (OS) data of mRNA expression of predicted target genes were collected from Kaplan-Meier plotter (http://www.kmplot.com/). The protein expression data were collected from Human Protein Atlas (https://www.proteinatlas.org). The data of pan cancer analysis of predicted target genes were collected from TIMER2.0 (http://timer.cistrome.org). Gene mutations of predicted target genes in BLCA and their OS and Progression Free Survival (PFS) were analyzed by cBioPortal (http://www.cbioportal.org). Finally, the drug sensitivity and resistance of TP53 mutation in BLCA were analyzed by Genomics of Drug Sensitivity in Cancer (https://www.cancerrxgene.org).

### Ethics approval

The study protocols were conducted according to the principles of the Declaration of Helsinki and were approved by the Scientific and Medical Ethical Committee of the Second Affiliated Hospital of Shantou University Medical College.

## RESULTS

### Over-expression of different miRNA in BLCA patients

**Figure 1:**
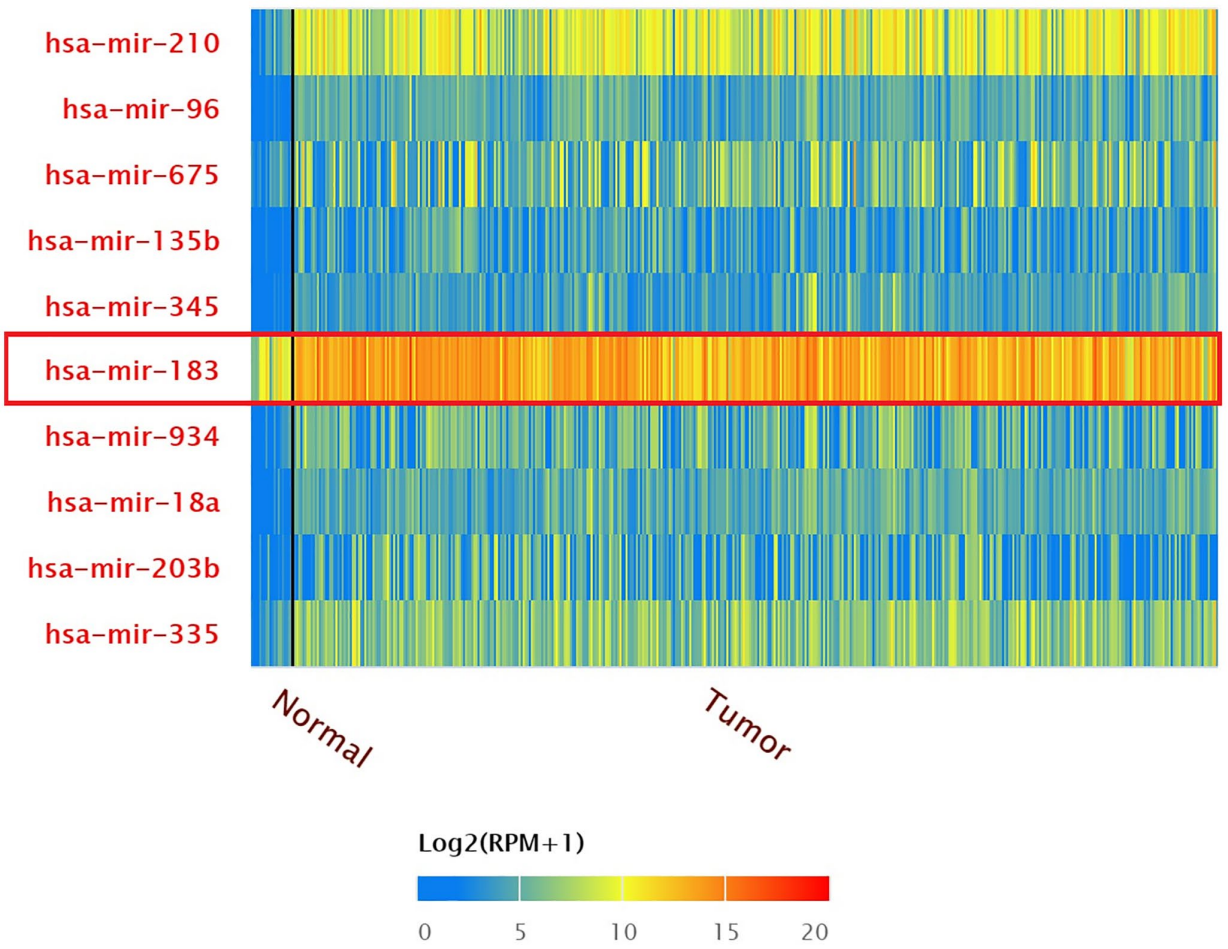
Top 10 over-expressed miRNAs in BLCA.

**Figure 2:**
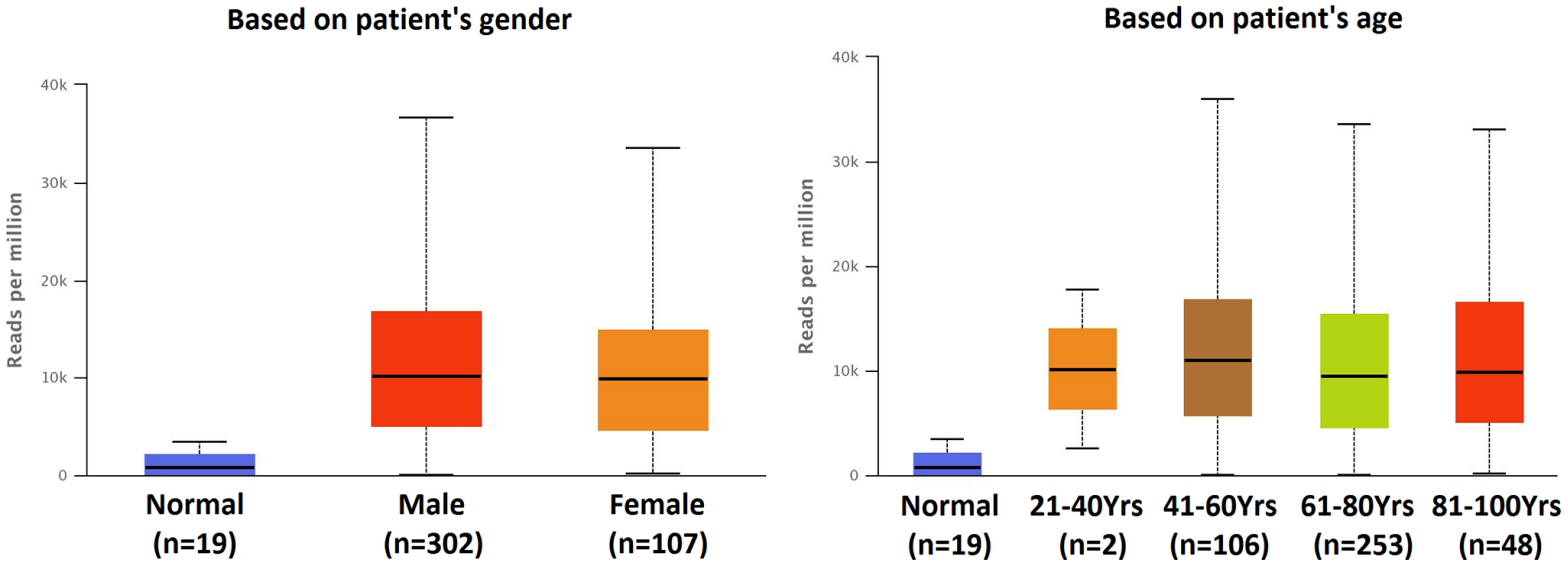
Expression of hsa-mir-183 in BLCA based on patient’s gender and age.

The result of top 10 over-expressed miRNA in BLCA patients were shown by TCGA miRNA analysis (Data from UALCAN) (Figure 1). The expression of hsa-mir-183 in patients with BLCA were significantly increased compared to normal tissue (*P* = 1.62E-12). The expression of hsa-mir-183 were also significantly increased based on patient’s gender or age (Figure 2). There were significant differences in the Comparison of Normal-vs-Male (*P* = 1.62E-12), Normal-vs-Female (*P* = 1.63E-8), Normal-vs-Age (41–60 Yrs) (*P* < 1E-12), Normal-vs-Age (61–80 Yrs) (*P* < 1E-12) and Normal-vs-Age (81–100 Yrs) (*P* = 7.55E-9).

### Expression of predicted target genes of hsa-mir-183 in BLCA patients

**Figure 3:**
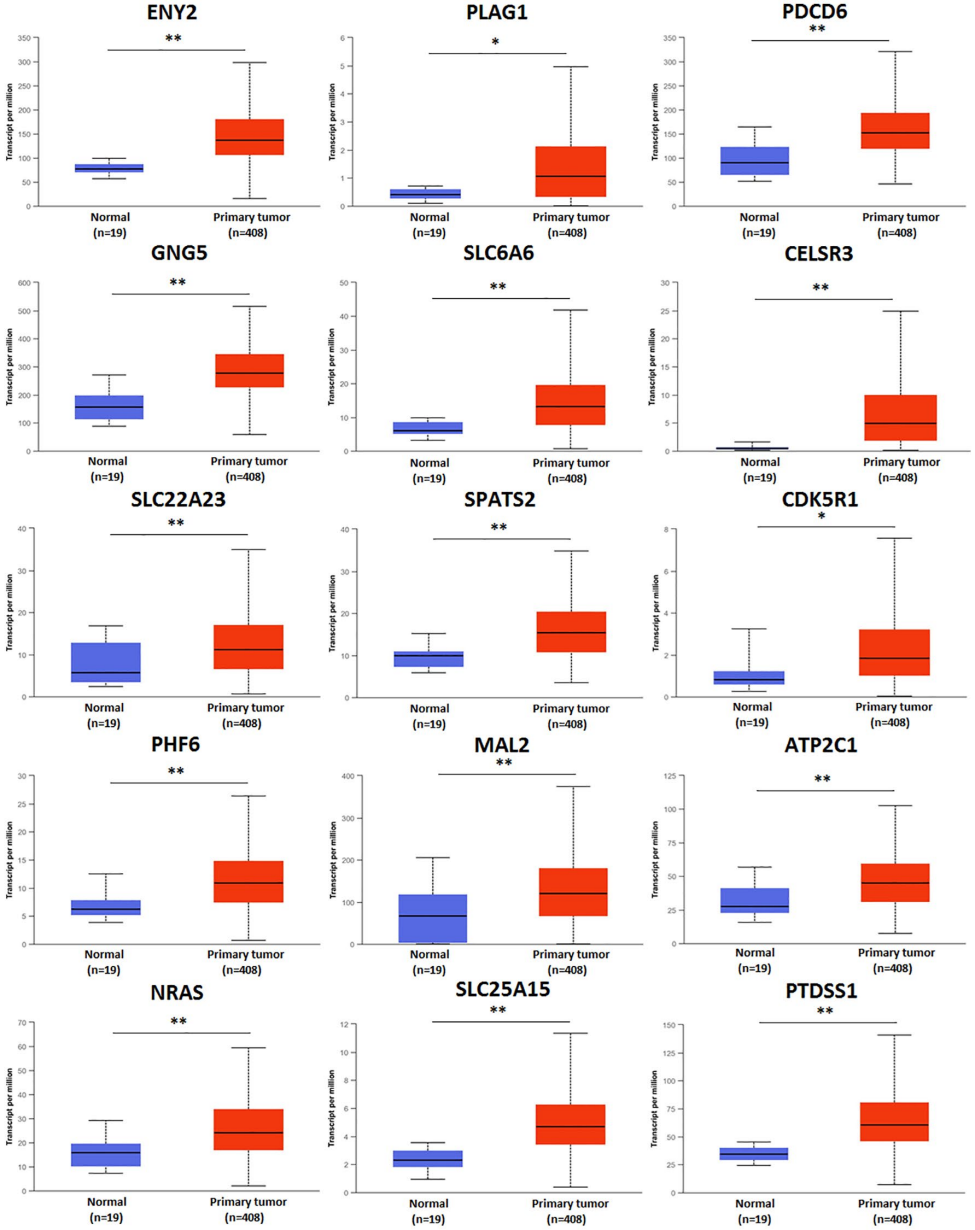
**Transcriptional expression of predicted target genes of hsa-mir-183 in BLCA based on TCGA sample types.** (^**^P < 0.001, ^*^P < 0.01).

There were 247 predicted genes of hsa-mir-183 (Data from TargetScan, microRNA.org and miRDB), including 15 up-regulated genes, 74 down-regulated genes and 158 unchanged genes in BLCA. 15 up-regulated genes were selected to study in our research (Table 1). The expression of all up-regulated predicted genes of has-mir-183 in patients with BLCA were significantly increased compared to normal tissue (Figure 3).

**Table 1:** Up-expressed predicted target genes of hsa-mir-183 in BLCA (TargetScan, microRNA.org and miRDB).

**Target gene**	**Description**	**Expression in BLCA**
ENY2	ENY2, transcription and export complex 2 subunit	Up-expression
PLAG1	PLAG1 zinc finger	Up-expression
PDCD6	programmed cell death 6	Up-expression
GNG5	G protein subunit gamma 5	Up-expression
SLC6A6	solute carrier family 6 member 6	Up-expression
CELSR3	cadherin EGF LAG seven-pass G-type receptor 3	Up-expression
SLC22A23	solute carrier family 22 member 23	Up-expression
SPATS2	spermatogenesis associated serine rich 2	Up-expression
CDK5R1	cyclin dependent kinase 5 regulatory subunit 1	Up-expression
PHF6	PHD finger protein 6	Up-expression
MAL2	mal, T cell differentiation protein 2	Up-expression
ATP2C1	ATPase secretory pathway Ca2+ transporting 1	Up-expression
NRAS	NRAS proto-oncogene, GTPase	Up-expression
SLC25A15	solute carrier family 25 member 15	Up-expression
PTDSS1	phosphatidylserine synthase 1	Up-expression

### Predicted functions and pathways of up-expressed predicted target genes of hsa-mir-183 and their 10 frequently altered neighbor genes

**Figure 4:**
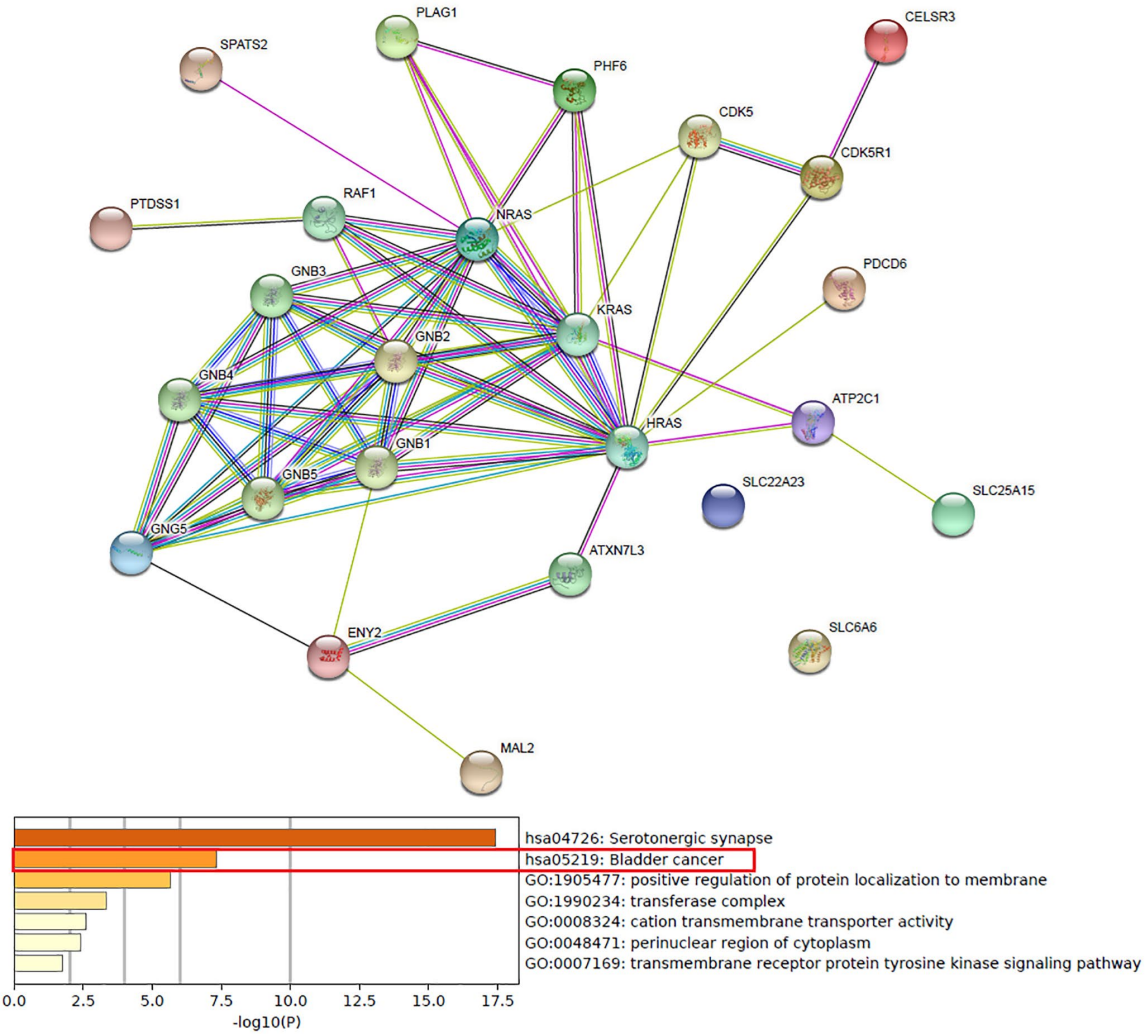
Predicted functions and pathways of up-expressed predicted target genes of hsa-mir-183 and their 10 frequently altered neighbor genes (Metascape).

Functions and pathways of up-expressed predicted target genes of hsa-mir-183 and their 10 frequently altered neighbor genes were analyzed by GO and KEGG in Metascape. As the Enrichment heatmap showed that pathways included hsa04726: Serotonergic synapse, hsa05219: Bladder cancer, GO:0007169: transmembrane receptor protein tyrosine kinase signaling pathway, GO:0048471: perinuclear region of cytoplasm, GO:0008324: cation transmembrane transporter activity, GO:1990234: transferase complex and GO:1905477: positive regulation of protein localization to membrane (Figure 4).

### Prognostic value of mRNA expression of up-expressed predicted target genes of hsa-mir-183 in BLCA patients

**Figure 5:**
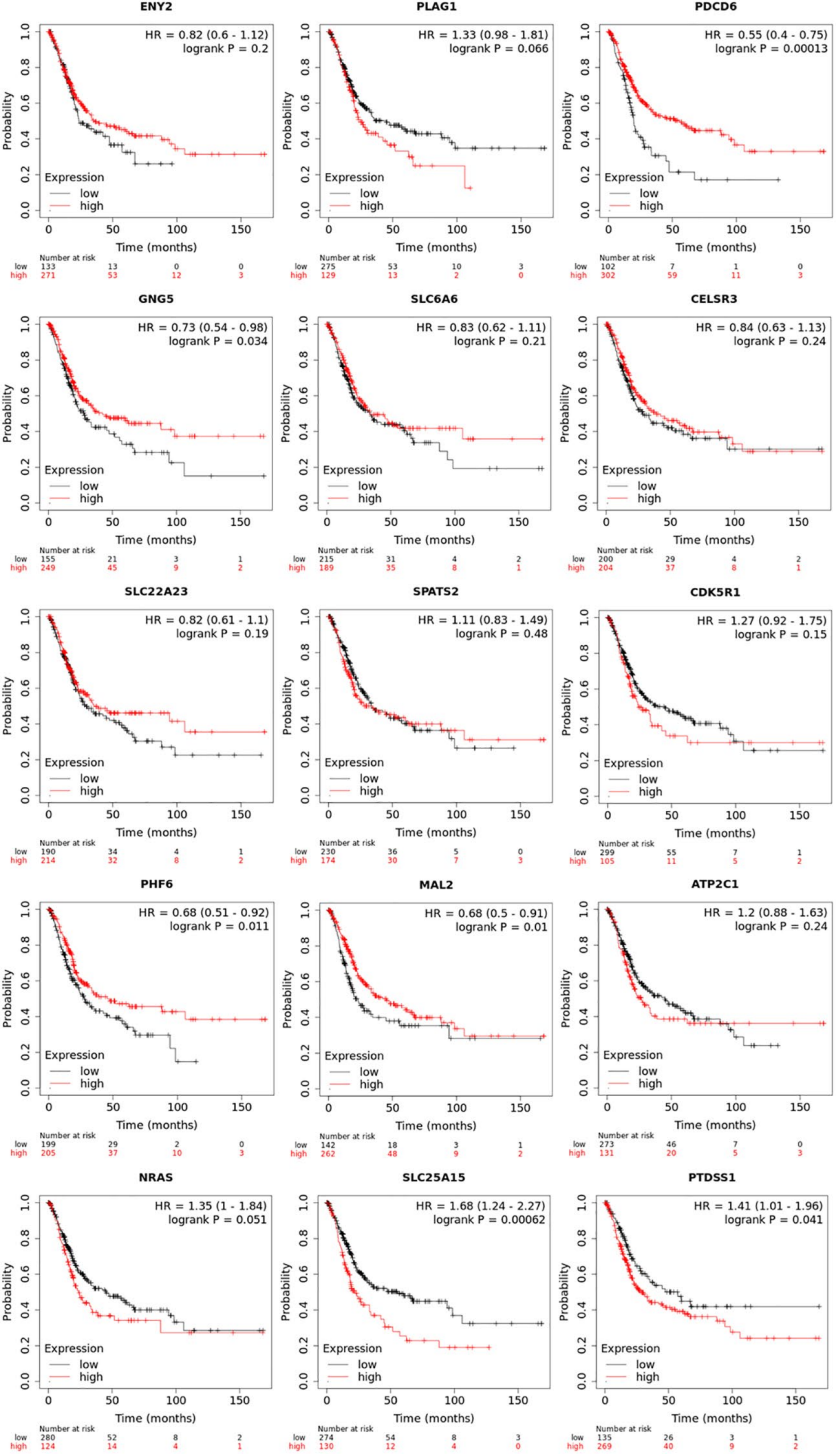
Prognostic value of transcriptional expression of up-expressed predicted target genes of hsa-mir-183 in BLCA (Kaplan-Meier Plotter).

Prognostic value of mRNA expression of up-expressed predicted target genes of hsa-mir-183 were analyzed by Kaplan-Meier plotter (Figure 5). It was showed that mRNA expressions of PDCD6, GNG5, PHF6, MAL2, SLC25A15 and PTDSS1 were significantly associated with BLCA patients’ prognosis. According to the database analysis form Kaplan-Meier plotter, PDCD6 (HR = 0.55, 95% CI: 0.4–0.75, and *P* = 0.00013), GNG5 (HR = 0.73, 95% CI: 0.54–0.89, and *P* = 0.034), PHF6 (HR = 0.68, 95% CI: 0.51–0.92, and *P* = 0.011) and MAL2 (HR = 0.68, 95% CI: 0.5–0.91, and *P* = 0.01) seem to be the favorable factor for BLCA patients’ prognosis. SLC25A15 (HR = 1.68, 95% CI: 1.24–2.27, and *P* = 0.00062) and PTDSS1 (HR = 1.41, 95% CI: 1.01–1.96, and *P* = 0.041) more likely to be the unfavorable factor for BLCA patients’ prognosis. In addition, ENY2, PLAG1, SLC6A6, CELSR3, SLC22A23, SPATS2, CDK5R1, ATP2C1 and NRAS were not prognostic in BLCA patients.

### Protein expression and pan cancer analysis of predicted target genes (PDCD6, GNG5, PHF6, MAL2, SLC25A15 and PTDSS1) in BLCA patients

**Figure 6:**
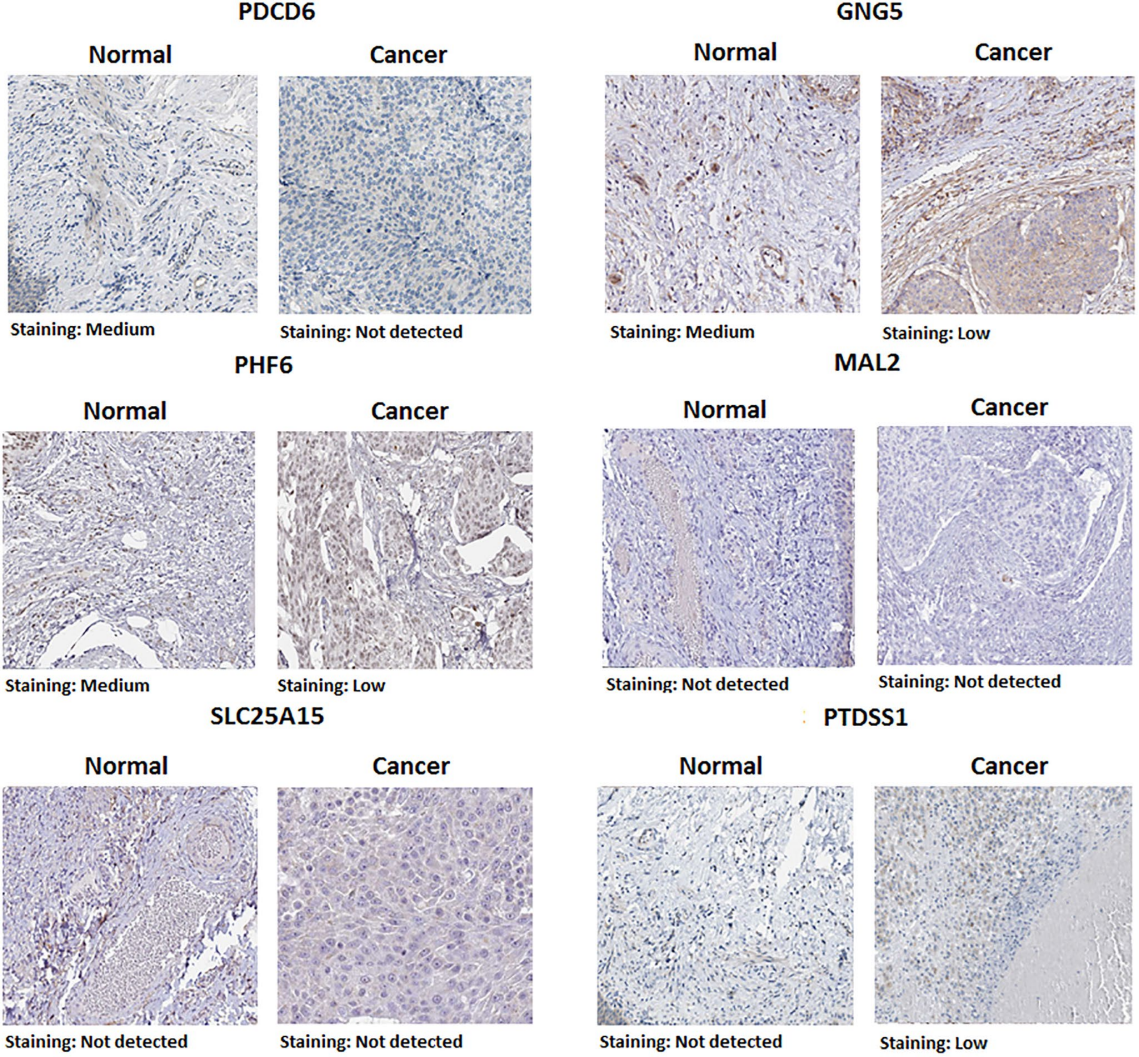
Representative immunohistochemistry images of predicted target genes (PDCD6, GNG5, PHF6, MAL2, SLC25A15 and PTDSS1) in normal urinary bladder tissues and BLCA tissues (Human Protein Atlas).

**Figure 7:**
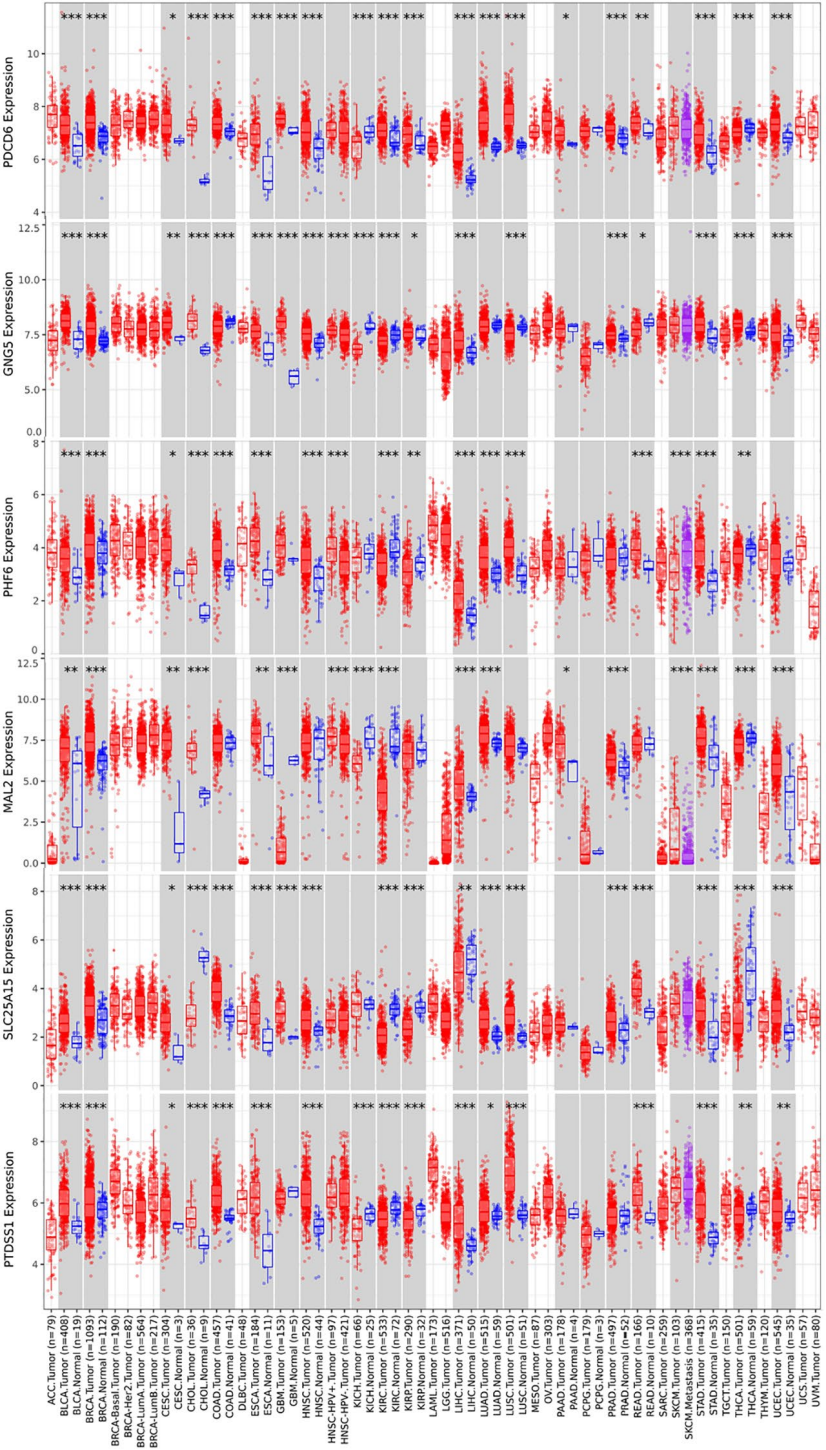
**Pan cancer analysis of predicted target genes (PDCD6, GNG5, PHF6, MAL2, SLC25A15 and PTDSS1) (TIMER).** (^***^P < 0.001, ^**^P < 0.01, ^*^P < 0.05).

Protein expression of predicted target genes (PDCD6, GNG5, PHF6, MAL2, SLC25A15 and PTDSS1) in BLCA were explored by Human Protein Atlas (Figure 6). The proteins of PDCD6, GNG5 and PHF6 were not or low expressed in BLCA tissues, whereas medium expressions of them were found in normal urinary bladder tissues. It is noteworthy that the protein expression of MAL2 and SLC25A15 could not be detected in both normal urinary bladder tissues and BLCA tissues. Additionally, the protein expression of PTDSS1 in tumor tissues was stronger than that in normal tissues. Furthermore, we performed a pan-cancer analysis of predicted target genes (PDCD6, GNG5, PHF6, MAL2, SLC25A15 and PTDSS1) by using TIMER (Figure 7).

### Relationship between mRNA expression of predicted target genes (PDCD6, GNG5, PHF6, MAL2, SLC25A15, PTDSS1) and clinicopathological parameters in BLCA patients

**Figure 8:**
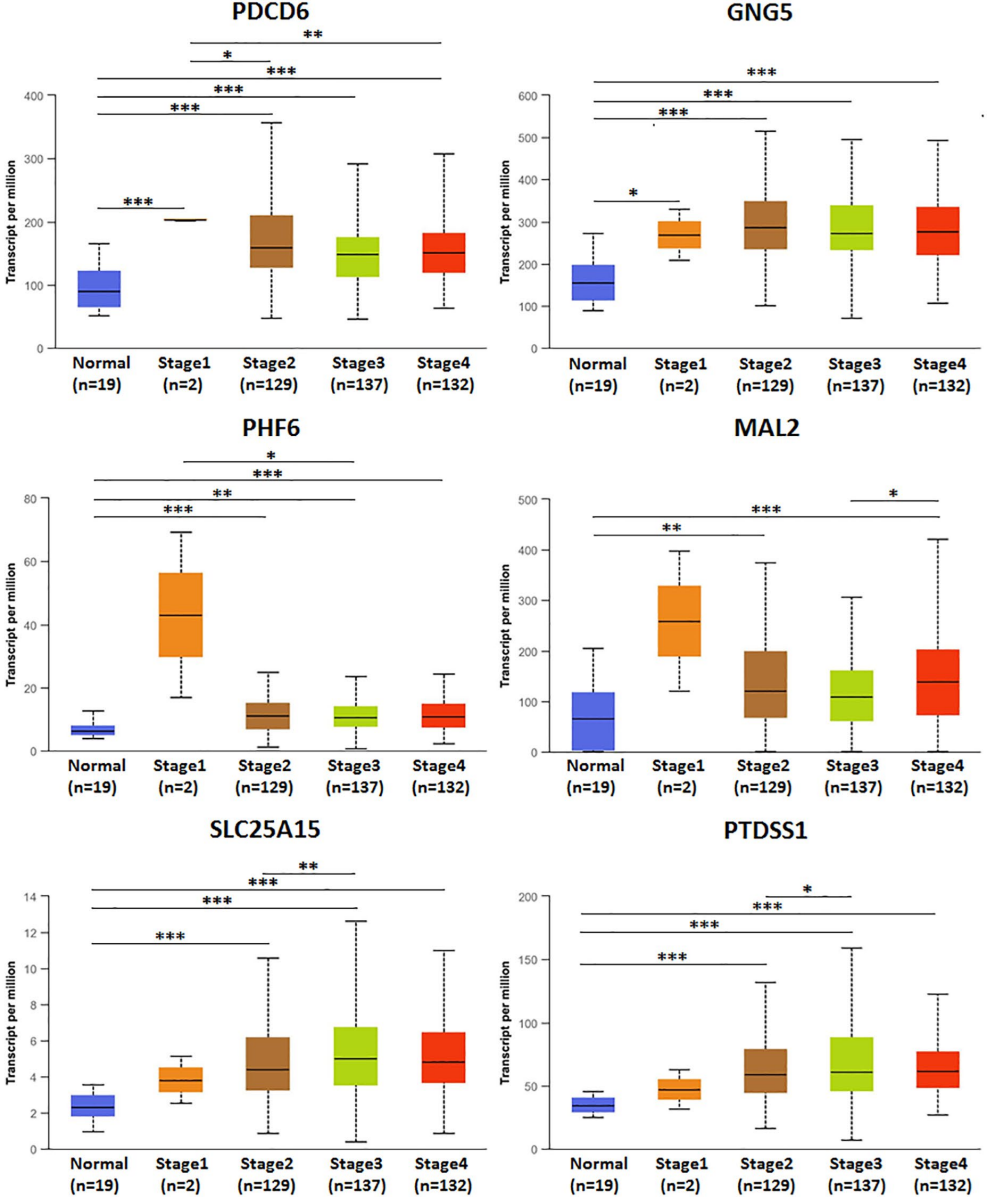
**Relationship between mRNA expression of predicted target genes (PDCD6, GNG5, PHF6, MAL2, SLC25A15, PTDSS1) and BLCA individual cancer stages.** (^***^P < 0.001, ^**^P < 0.01, ^*^P < 0.05).

**Figure 9:**
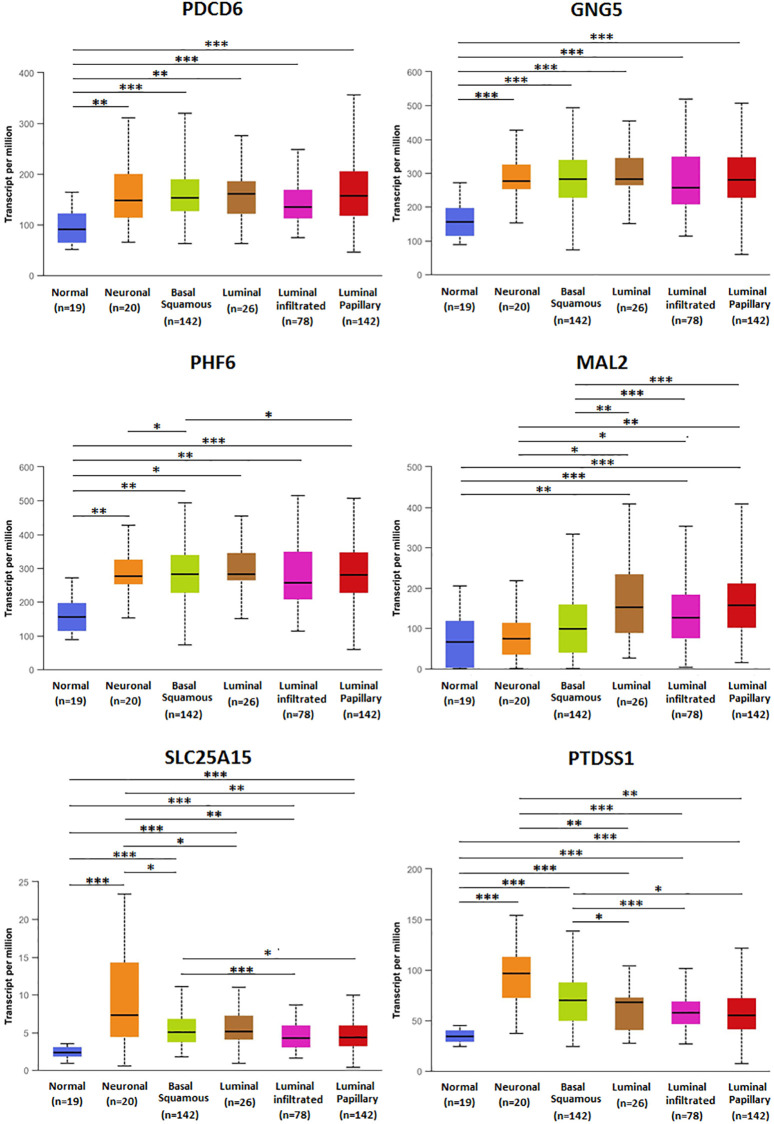
**Relationship between mRNA expression of predicted target genes (PDCD6, GNG5, PHF6, MAL2, SLC25A15, PTDSS1) and BLCA molecular subtypes.** (^***^P < 0.001, ^**^P < 0.01, ^*^P < 0.05).

The association of mRNA expression of predicted target genes (PDCD6, GNG5, PHF6, MAL2, SLC25A15, PTDSS1) with clinicopathological parameters of BLCA patients were analyzed by UALCAN, containing BLCA individual cancer stages and molecular subtypes (Figures 8 and 9). Figure 8 showed that the mRNA expression of predicted target genes (PDCD6, GNG5, PHF6, MAL2, SLC25A15, PTDSS1) were significantly correlated with BLCA individual cancer stages. PDCD6, GNG5, PHF6 and MAL2 as favorable factor for BLCA patients, the high mRNA expressions of them tended to be in stage 1 or 2, whereas the high mRNA expressions of unfavorable factor of SLC25A15 and PTDSS1 tended to be in stage 3 or 4. Similarly, Figure 9 showed that the mRNA expression of predicted target genes (PDCD6, GNG5, PHF6, MAL2, SLC25A15, PTDSS1) were significantly correlated with BLCA molecular subtypes. The high mRNA expressions of PDCD6, GNG5, PHF6 and MAL2 tended to be the molecular subtypes of luminal and luminal papillary, whereas the high mRNA expressions of SLC25A15 and PTDSS1 tended to be the molecular subtypes of neuronal and basal squamous.

### Gene mutations of predicted target genes (PDCD6, GNG5, PHF6, MAL2, SLC25A15, PTDSS1) in BLCA

**Figure 10:**
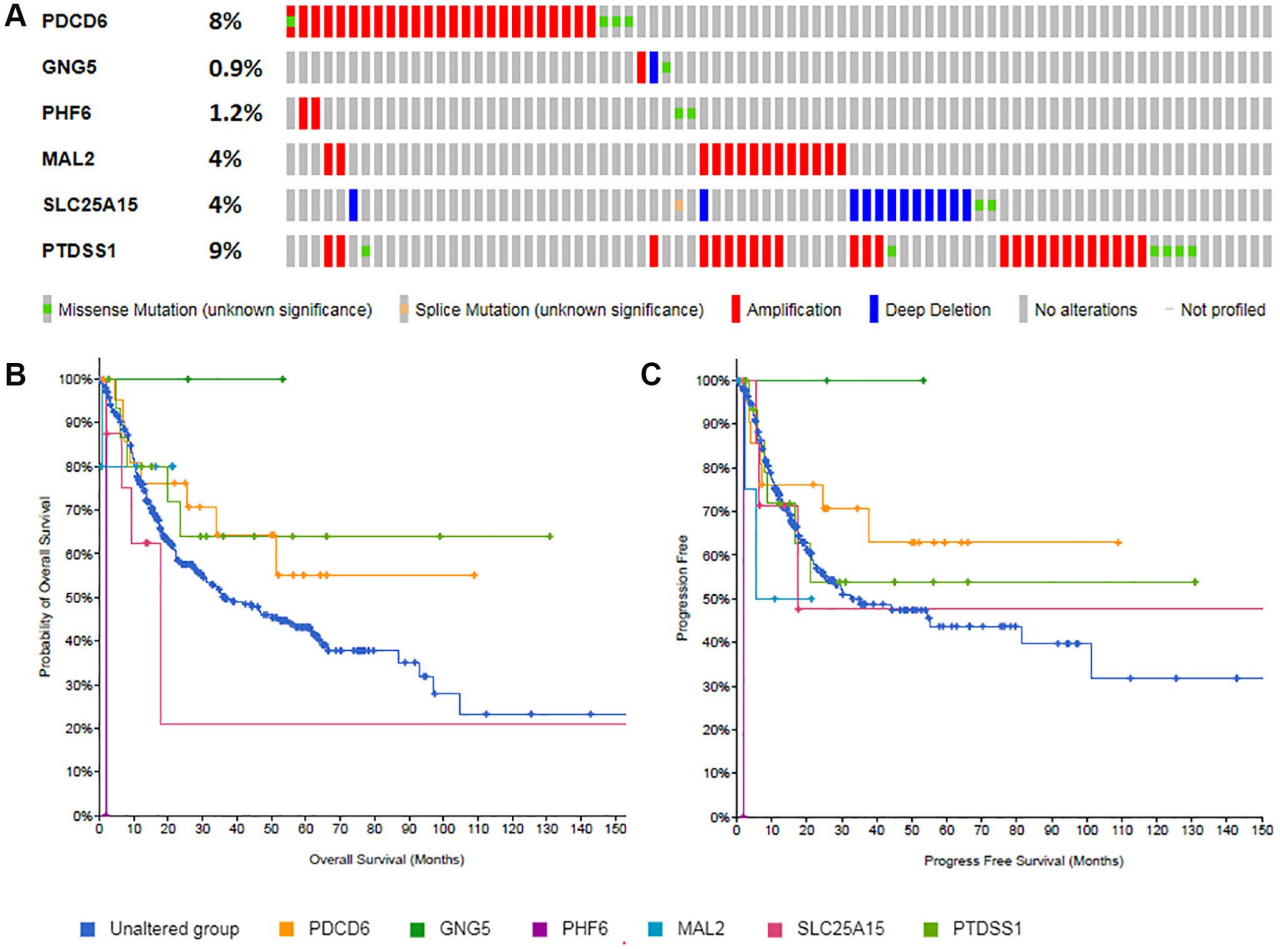
Gene mutations of predicted target genes (PDCD6, GNG5, PHF6, MAL2, SLC25A15, PTDSS1) and their association with OS and PFS in BLCA patients (**A**: Genetic alterations; **B**: Overall survival; **C**: Progress free survival).

Gene mutations of predicted target genes (PDCD6, GNG5, PHF6, MAL2, SLC25A15, PTDSS1) in BLCA and their OS and PFS were analyzed by cBioPortal (Figure 10). Figure 10A showed that PDCD6, MAL2, SLC25A15, PTDSS1 were prone to mutation in BLCA patients. Figure 10B and 10C revealed the association between the gene mutation and BLCA patients’ OS and DFS. In OS analyze, median months overall (95% CI) of unaltered group was 36.43, while it was significantly shorter in PHF6 (1.87) and SLC25A15 (17.62). Similarly, in PFS analyze, Median Months Progression Free (95% CI) of unaltered group was 32.58, while it was significantly shorter in PHF6 (1.87), MAL2 (5.52) and SLC25A15 (17.52).

### TP53 mutation of predicted target genes (PDCD6, GNG5, PHF6, MAL2, SLC25A15, PTDSS1) and its association with drug selection in BLCA

**Figure 11:**
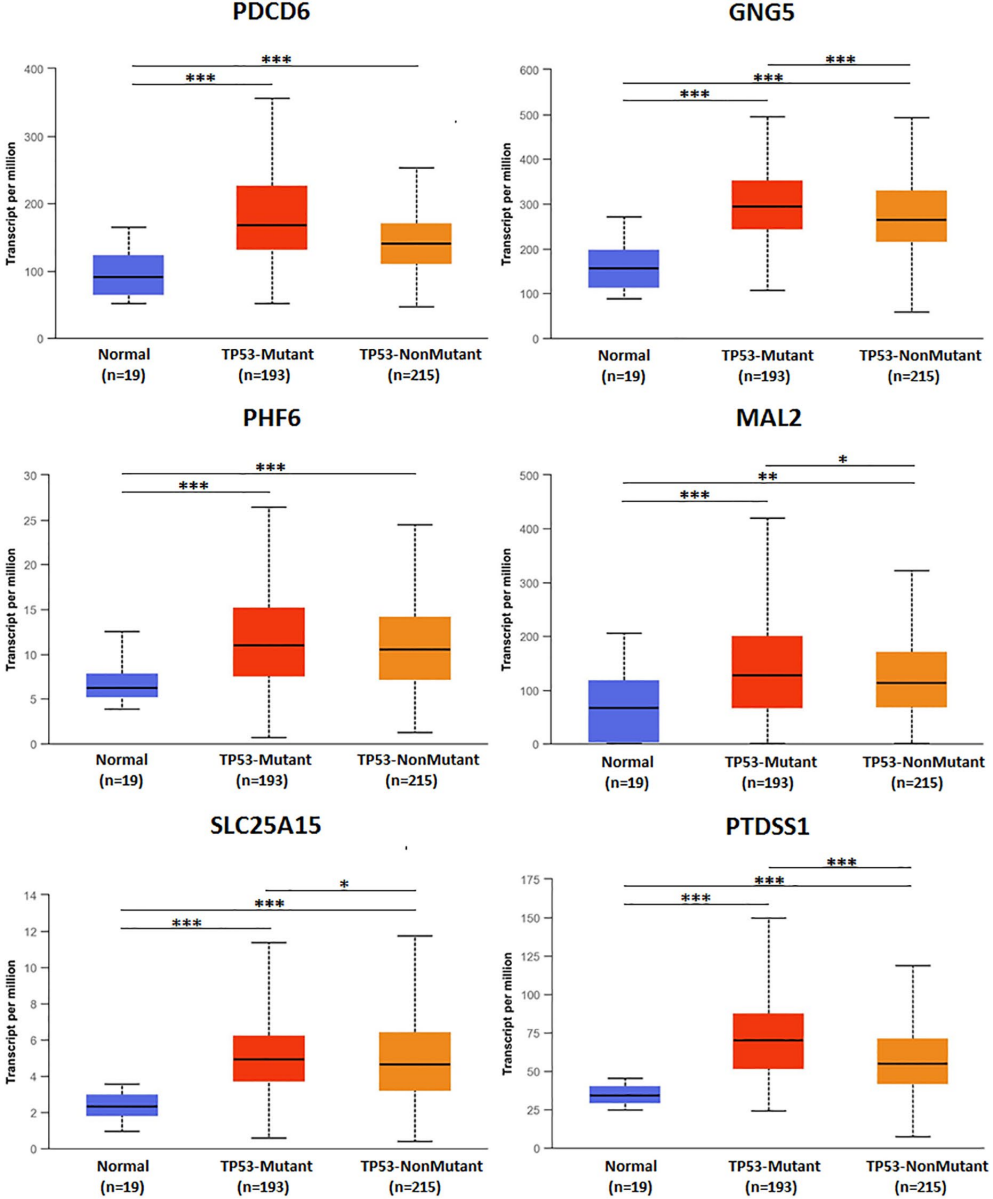
TP53 mutation of predicted target genes (PDCD6, GNG5, PHF6, MAL2, SLC25A15, PTDSS1) in BLCA.

**Figure 12:**
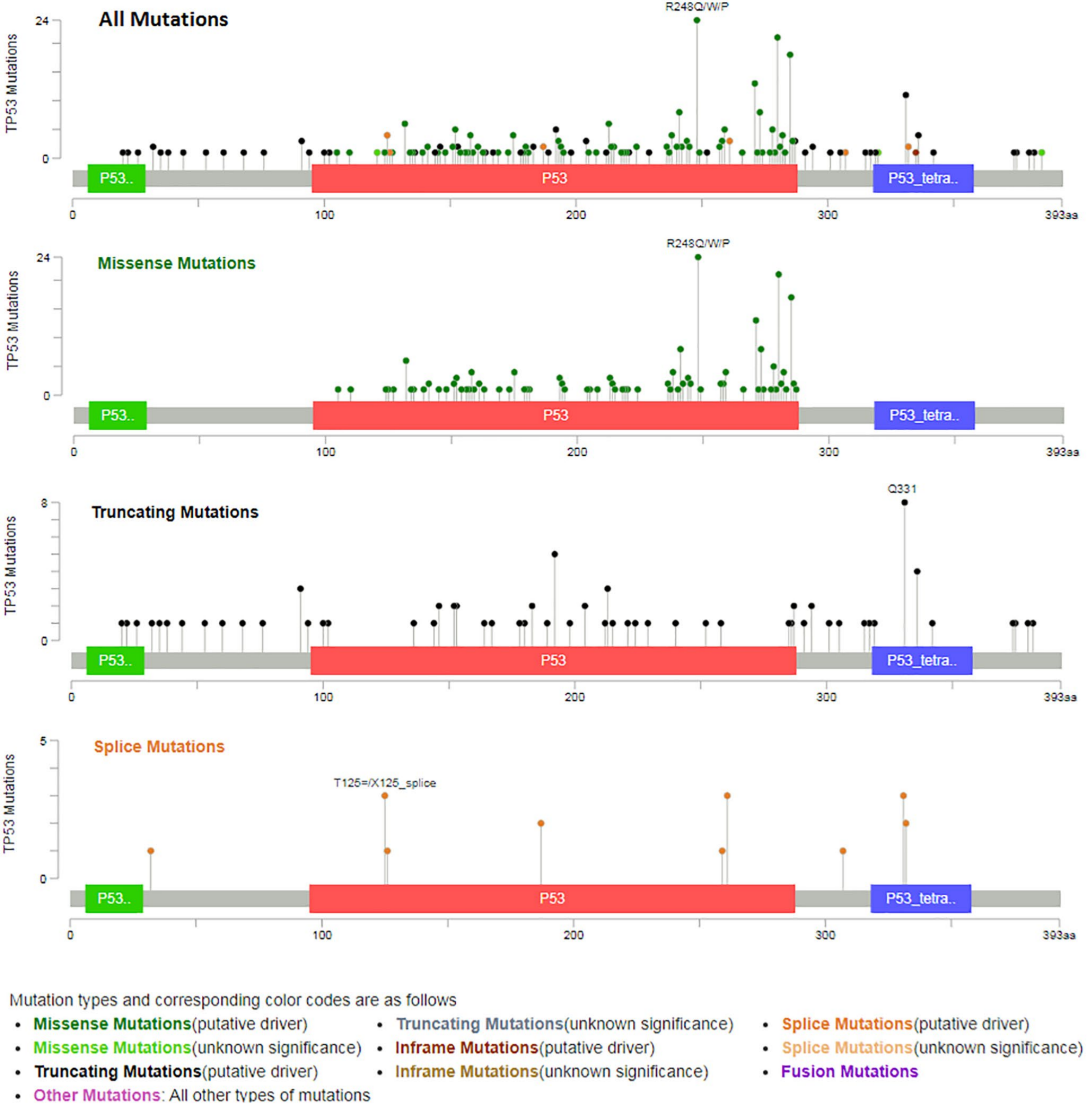
Types of TP53 mutation from bladder cancer (MSK/TCGA, 2020).

TP53 mutation of predicted target genes (PDCD6, GNG5, PHF6, MAL2, SLC25A15, PTDSS1) were analyzed by UALCAN. The expression of TP53 mutation of GNG5, MAL2, SLC25A15, PTDSS1 in patients with BLCA were significantly increased compared to non-mutation group, and all predicted target genes were markedly increased compared to normal group (Figure 11). In addition, types of TP53 mutation were analyzed by cBioPortal (Figure 12) from Bladder Cancer (MSK/TCGA, 2020). Mutation frequency of TP53 was 51.7% from NM_000546 | ENST00000269305 CCDS11118 | P53_HUMAN.

**Figure 13:**
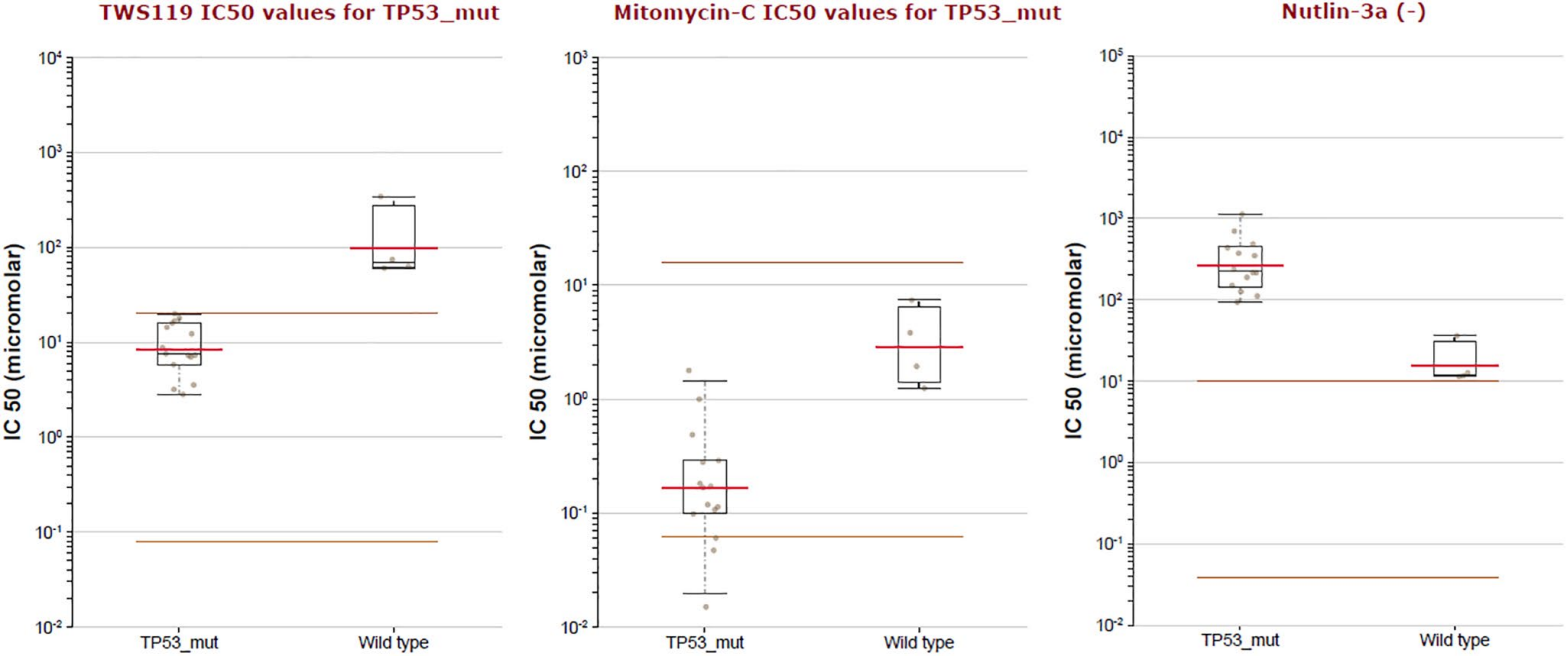
The IC50 of TWS119, Mitomycin-C and Nutlin-3a (−) in TP53 mutation of BLCA patients.

As was showed in Figure 12, TP53 driver had the highest probability of mutation, including Missense (197), Truncating (80) and Splice (17). Furthermore, the drug sensitivity and resistance of TP53 mutation in BLCA were analyzed by Genomics of Drug Sensitivity in Cancer (Figure 13). Nutlin-3a (−) drug target was MDM2 and Nutlin-3a (−) drug target pathway was p53 pathway in BLCA. As was showed in Figure 13 and Table 2, Nutlin-3a (−) had a significant increased resistance in TP53 mutation (*P* = 2.12e-06) of BLCA patients and the IC50 of Nutlin-3a (−) was significantly increased in TP53 mutation group (*P* < 0.001). TWS119 drug target was GSK3 and TWS119 drug target pathway was WNT signaling in BLCA. As was showed in Figure 13 and Table 2, TWS119 had a significant increased sensitivity in TP53 mutation (*P* = 5.95e-06) of BLCA patients and the IC50 of TWS119 was significant decreased in TP53 mutation group (*P* < 0.001). Mitomycin-C drug target was DNA crosslinker and Mitomycin-C drug target pathway was DNA replication in BLCA. As was showed in Figure 13 and Table 2, Mitomycin-C had a significant increased sensitivity in TP53 mutation (*P* = 0.000322) of BLCA patients and the IC50 of Mitomycin-C was significant decreased in TP53 mutation group (*P* = 0.001).

**Table 2:** Association between TP53 mutation and drug option of BLCA.

**Drug**	**Drug Target**	**IC50 effect**	**Effect size**	***P*-value**	**FDR%**	**Tissue**
TWS119	GSK3	Increased sensitivity	−3.63	5.95e-06	0.00714	BLCA
Mitomycin-C	DNA crosslinker	Increased sensitivity	−2.53	0.000322	0	BLCA
Nutlin-3a (−)	MDM2	Increased resistance	4.08	2.12e-06	0.00255	BLCA

## DISCUSSION

BLCA was the most common histopathological type of bladder cancer, and its occurrence and development were affected by multiple complex factors such as gene transcription, gene regulation, gene mutation and epigenetic inheritance [14, 15]. Each miRNA had a series of related target genes, and abnormal expression of miRNA often leaded to abnormal transcription of target genes, which was related to the occurrence of various cancers [16]. Although hsa-mir-183 have been reported to play an essential role in liver cancer, lung cancer and other tumors, the specific role of hsa-mir-183 and its predicted target genes in BLCA remain to be explored. In our study, the transcriptional expressions, mutations and Prognosis of hsa-mir-183 and its predicted target genes in BLCA have been elucidated.

Our study found that up-expressed hsa-mir-183 and over transcriptional expressions of hsa-mir-183 predicted target genes (ENY2, PLAG1, PDCD6, GNG5, SLC6A6, CELSR3, SLC22A23, SPATS2, CDK5R1, PHF6, MAL2, ATP2C1, NRAS, SLC25A15 and PTDSS1) were observed in BLCA patients. Moreover, predicted functions and pathways of up-expressed predicted target genes of hsa-mir-183 and their 10 frequently altered neighbor genes indicated a link with bladder cancer. Besides, high transcriptional expressions of PDCD6, GNG5, PHF6 and MAL2 were markedly relevant to favorable OS in BLCA patients, whereas high transcriptional expression of SLC25A15 and PTDSS1 were markedly relevant to unfavorable OS in BLCA patients. Additionally, ENY2, PLAG1, SLC6A6, CELSR3, SLC22A23, SPATS2, CDK5R1, ATP2C1 and NRAS were not prognostic in BLCA patients. However, protein expression of predicted target genes (PDCD6, GNG5, PHF6, MAL2, SLC25A15 and PTDSS1) were inconsistent with the transcriptional expression. The mRNA levels were significantly increased than that observed in protein levels. There are two possible explanations for this phenomenon. miRNA do not limit gene translation but lead to rapid destruction of newborn peptides [17]. Alternatively, miRNA may negatively affect the initiation, elongation and termination of transcription, while the number of ribosomes remains unchanged, resulting in a decreased rate of protein completion [17]. Multivariate analysis indicated that transcriptional expression of predicted target genes (PDCD6, GNG5, PHF6, MAL2, SLC25A15 and PTDSS1) were significantly correlated with BLCA individual cancer stages and molecular subtypes. Furthermore, high mutation rates of PDCD6, MAL2, SLC25A15 and PTDSS1 were observed in BLCA patients and some genetic alteration were significantly relevant to OS and PFS. Finally, TP53 mutation of predicted target genes (PDCD6, GNG5, PHF6, MAL2, SLC25A15 and PTDSS1) has guiding significance for drug selection in BLCA.

### PDCD6

Programmed cell death 6, can encode the calcium binding protein, which is belonging to the penta-EF-hand protein family. High expression of PDCD6 was relevant to unfavorable prognosis in colorectal cancer patients via activating c-Raf, MEK and ERK pathway [18]. Inhibition of PDCD6 expression can reduce the rate of tumor metastasis [19]. PDCD6 can induce migration and invasion of tumor cell in ovarian cancer causing unfavorable clinical outcome to patients [20]. However, the expression of GG genotype of PDCD6 has been reported to reduce the risk of lung cancer [21]. Similarly, up-expressed PDCD6 played the role of inhibiting cell growth in cervical carcinoma cells [22]. These results suggested that PDCD6 may play different roles in tumorigenesis and prognosis. In our study, the transcriptional expression of PDCD6 was significantly increased in BLCA and suggested a favorable prognosis. The function of MAPK pathway is to induce cell hyperplasia and invasion, but it can also increase the occurrence of cell apoptosis or inhibit cell invasion. Different effects of PDCD6 predominantly depend on the signal strength and the microenvironment of abnormal signal activation [23]. In addition, the downstream MAPK signal is mainly regulated by the upstream c-Raf signal. It has been proved that PDCD6-c-Raf-MAPK pathway exists in tumors in recent research [18]. Therefore, we speculated that tumor microenvironment or signal specificity lead to different roles of PDCD6 in tumors.

### GNG5

G protein subunit gamma 5, can regulate the information from a variety of receptors. High expression of GNG5 was relevant to unfavorable prognosis in gliomas [24]. At present, there are few studies on GNG5 in other tumors. It was reported that GNG5 may regulate the occurrence and development of tumors by stimulating the PI3K/Akt signal pathway [25]. Akt is activated by upstream PI3K, leading to phosphorylation, which finally promotes tumor cell growth. To our knowledge, this is the first study to explore the role of GNG5 in BLCA. Transcriptional expression of GNG5 was found to be significantly associated with BLCA individual cancer stages, BLCA molecular subtypes and OS. Of note, higher expression of GNG5 indicated a longer possibility of survival. Therefore, GNG5 may act as an advanced indicator of the stages, types and prognosis for BLCA which has not been reported in previous researches.

### PHF6

PHD finger protein 6, has a function of transcriptional regulation by encoding domain with 2 PHD-type zinc fingers in protein. The high expression of PHF6 can play a role of tumor suppressor in patients with acute lymphoblastic leukemia and as a favorable prognostic factor [26–28]. However, other study has come up with an inconsistent view that PHF6 was involved in proliferation, migration, apoptosis and metabolism in hepatocellular carcinoma and its deficiency can inhibit above mentioned processes in tumor cells [29]. Wang et al., found that PHF6-null can inhibit cell proliferation and arrest cells in the G(2)/M phase, suggesting that loss of PHF6 may result in accumulation of DNA damage [30]. Hsu et al., reported that the deficiency of PHF6 can enhance cell self-renewal and carcinogenic potential [31]. Together, these findings indicated that whether PHF6 was a tumor suppressor or a tumor protein may depend on the specific context in which it acted. In our study, high transcriptional expression of PHF6 was significantly related to high probability of survival in BLCA which were consistent with the research results in acute lymphoblastic leukemia.

### MAL2

Mal, T cell differentiation protein 2, can encode multitransmembrane protein, which is a member of MAL proteolipid family. MAL2 was over-expressed in many human cancers, including breast, stomach, liver, pancreatic, colon, kidney, prostate and ovarian cancers [32–36]. Zheng et al., found that MAL2 was markedly relevant to grade, stage and the Gleason score of prostate cancer and it can promote tumor progression via the Notch pathway [33]. Fang et al., showed that Inhibition of MAL2 expression can significantly increase the cytotoxicity of CD8^+^ T lymphocytes, thereby inhibiting the growth and metastasis of breast cancer [35]. Zhang et al., reported that overexpressed MAL2 can phosphorylate ERK1/2, which may promote pancreatic cancer progression [37]. In BLCA patients, MAL2 was also highly expressed, but it was associated with a good prognosis. At present, the specific signaling pathway that MAL2 participates in bladder cancer is not clear, so we speculate that up-expressed MAL2 may not be the initiating factor of tumorigenesis, but may lead to changes in cell morphology, signal transduction, migration and metabolism by altering protein distribution, thus promoting tumor transformation [38], which is bidirectional.

### SLC25A15

Solute carrier family 25 member 15, can encode the protein transporter ornithine, which is a member of mitochondrial carrier family. Ji et al., found that SLC25A15 was up-expressed in melanoma patients and was negatively associated with OS and DFS [39]. SLC25A15 was involved in the regulation of cell proliferation and apoptosis in cells and over-expression can reverse the role of tumor suppressor genes in prostate cancer [40]. At present, most of the studies on SLC25A15 are focused on hyperornithinemia-hyperammonemia-homocitrullinuria [41], and there are few reports on tumor. SLC25A15 is primarily responsible for transporting ornithine from the cytoplasm to the mitochondrial matrix, which is an important step in regulating the urea cycle [42]. Whether SLC25A15 has other potential signaling pathways involved in tumor progression. However, by comparing recent researches with our present study, it can be preliminarily speculated that SLC25A15 is an unfavorable prognostic signal for BLCA, which may act as a new biomarker to assist the diagnosis and prognosis of BLCA.

### PTDSS1

Phosphatidylserine synthase 1, has a function to promote the synthesis of phosphatidylserine. Li et al., found that over-expression of PTDSS1 in astroglioma involved in lipid metabolism [43]. Wang et al., shown that PTDSS1 was an oncogene of lung adenocarcinoma, and overexpression was significantly associated with low survival [44]. PTDSS1 can participate in the biosynthesis of phosphatidylserine by directing the synthesis of a key enzyme, phosphatidylserine synthase, which was an important part of phosphatidylserine metabolism [44]. PTDSS1-mediated phosphatidylserine signaling has been shown to be severely disrupted in metabolism of tumor cell and pathogenesis of autoimmune disorders [45]. Tumor progression and metastasis are greatly influenced by the interaction between tumor cells and their living microenvironment. TAM receptors exist on the surface of tumor cells, and an environment rich in phosphatidylserine can provide a platform for activation of TAM receptors [46]. In our study, high transcriptional expression of PTDSS1 was significantly related to low probability of survival in BLCA which indicated that PTDSS1-mediated phosphatidylserine signaling was one of the pathogenesis of BLCA.

## CONCLUSION

To sum up, we concluded that PDCD6, GNG5, PHF6, MAL2, SLC25A15 and PTDSS1 could be the advanced independent indicators for prognosis of BLCA patients, and TP53-mutation might be a biomarker for drug option in BLCA patients.
